# Multimodality imaging findings of cardiac neuroendocrine tumour metastasis

**DOI:** 10.1093/ehjcr/ytad587

**Published:** 2023-11-20

**Authors:** Moezedin Javad Rafiee, Marc Hickeson, Michael Chetrit

**Affiliations:** Department of Diagnostic Radiology, McGill University Health Centre, 1001 Blvd, Decarie, Montreal, H4A 3J1 Québec, Canada; Department of Nuclear Medicine, McGill University Health Centre, Montreal, QC, Canada; Department of Diagnostic Radiology, McGill University Health Centre, 1001 Blvd, Decarie, Montreal, H4A 3J1 Québec, Canada; Division of Cardiology, Department of Medicine, McGill University Health Centre, Montreal, QC, Canada

**Figure:**
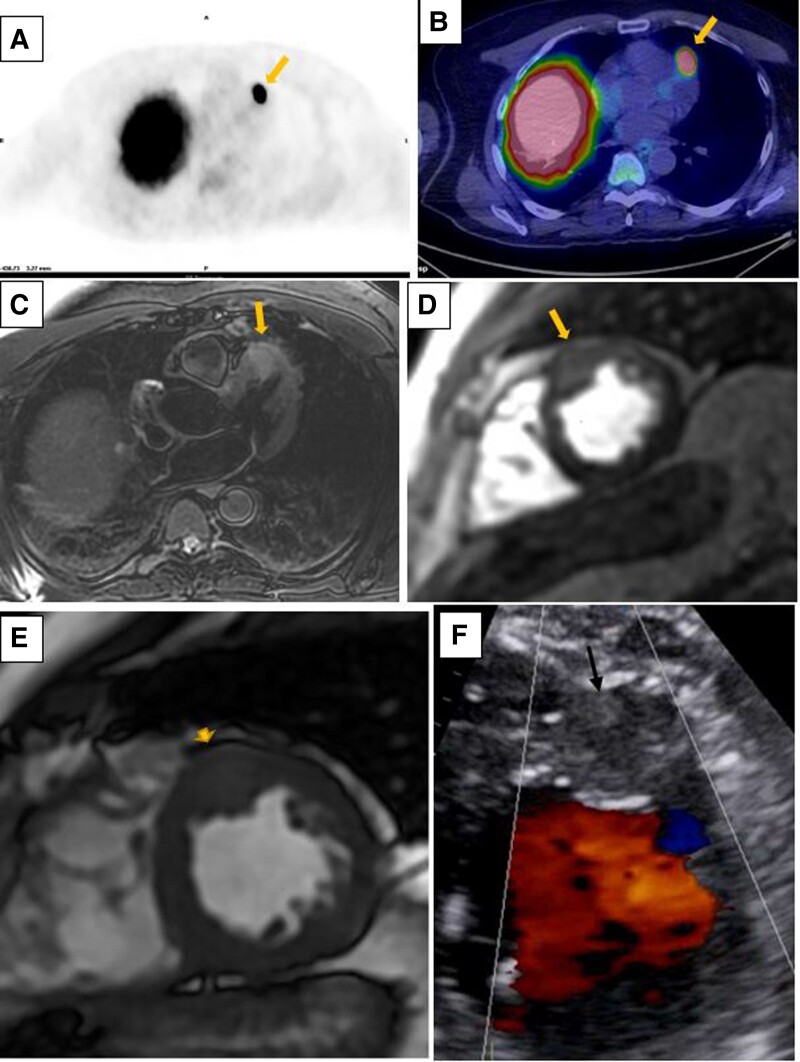


A 72-year-old man, diagnosed with well-differentiated neuroendocrine tumour (NET) of the ileum, was referred to our hospital for staging and treatment.

At initial staging using ^68^Ga-DOTATATE positron emission tomography (PET)/computed tomography (CT) scan, multifocal DOTATATE-avid lesions were found in the skeleton, liver, and the anterior LV wall (*A* and *B*, arrow). Biopsy proved NET hepatic metastasis. Further evaluation with transthoracic echocardiography revealed a 22.5 × 21.1 mm lesion in the mid anterior LV wall with vascularity (*F*, arrow) without thickening or restriction of the tricuspid and pulmonary valves to suggest carcinoid heart syndrome.

Subsequent cardiac magnetic resonance (CMR) imaging revealed normal biventricular size and global systolic function. An intra-myocardial solid lesion, measuring 21 × 20 × 14 mm, hyperintense on T2-W axial (*C*, arrow), isointense on short axis SSFP (*E*, arrowhead), and hypervascular on first-pass perfusion images (*D*, arrow), was demonstrated in the mid LV anterior wall. Given the concordance of findings from PET and CMR, a diagnosis of cardiac NET metastasis was confirmed.

Neuroendocrine tumours with heart metastasis are very rare occurring in only 1.75% cases although are clinically relevant. The left ventricle is the most commonly involved cardiac chamber. While echocardiography has a low sensitivity for detecting cardiac NET metastasis, both CMR and ^68^Ga-DOTATATE PET have excellent accuracy for its diagnosis and monitoring. Interestingly, NET cardiac metastasis and carcinoid heart disease are two distinct entities; they coexist in 8% of reported cases. Images on this report would help identifying cardiac NET metastasis.

**Consent:** The authors confirm that written consent for the submission and publication of this case, including images, has been obtained from the patient and his parents in line with the Committee on Publication Ethics (COPE) guidance.

**Funding:** None declared.

## Data Availability

The data underlying this article will be shared on reasonable request to the corresponding author.

